# Mx Is Not Responsible for the Antiviral Activity of Interferon-α against Japanese Encephalitis Virus

**DOI:** 10.3390/v9010005

**Published:** 2017-01-10

**Authors:** Jing Zhou, Shi-Qi Wang, Jian-Chao Wei, Xiao-Min Zhang, Zhi-Can Gao, Ke Liu, Zhi-Yong Ma, Pu-Yan Chen, Bin Zhou

**Affiliations:** 1 Key Laboratory of Animal Diseases Diagnosis and Immunology, Ministry of Agriculture, College of Veterinary Medicine, Nanjing Agricultural University, Nanjing 210095, China; 2015107081@njau.edu.cn (J.Z.); 15150560620@163.com (S.-Q.W.); xiaomin107228@126.com (X.-M.Z.); 2014107082@njau.edu.cn (Z.-C.G.); puyanchennj@163.com (P.-Y.C.); 2 Shanghai Veterinary Research Institute, Chinese Academy of Agricultural Science, Shanghai 200241, China; weijianchao@shvri.ac.cn (J.-C.W.); liuke@shvri.ac.cn (K.L.); zhiyongma@shvri.ac.cn (Z.-Y.M.)

**Keywords:** Mx1, Mx2, interferon-α (IFNα), Japanese encephalitis virus (JEV), antivirus, Brefeldin A (BFA)

## Abstract

Mx proteins are interferon (IFN)-induced dynamin-like GTPases that are present in all vertebrates and inhibit the replication of myriad viruses. However, the role Mx proteins play in IFN-mediated suppression of Japanese encephalitis virus (JEV) infection is unknown. In this study, we set out to investigate the effects of Mx1 and Mx2 expression on the interferon-α (IFNα) restriction of JEV replication. To evaluate whether the inhibitory activity of IFNα on JEV is dependent on Mx1 or Mx2, we knocked down Mx1 or Mx2 with siRNA in IFNα-treated PK-15 cells and BHK-21 cells, then challenged them with JEV; the production of progeny virus was assessed by plaque assay, RT-qPCR, and Western blotting. Our results demonstrated that depletion of Mx1 or Mx2 did not affect JEV restriction imposed by IFNα, although these two proteins were knocked down 66% and 79%, respectively. Accordingly, expression of exogenous Mx1 or Mx2 did not change the inhibitory activity of IFNα to JEV. In addition, even though virus-induced membranes were damaged by Brefeldin A (BFA), overexpressing porcine Mx1 or Mx2 did not inhibit JEV proliferation. We found that BFA inhibited JEV replication, not maturation, suggesting that BFA could be developed into a novel antiviral reagent. Collectively, our findings demonstrate that IFNα inhibits JEV infection by Mx-independent pathways.

## 1. Introduction

Japanese encephalitis virus (JEV)—a member of the genus *Flavivirus* within the family Flaviviridae—causes serious epidemics in tropical and subtropical areas with a high mortality rate of approximately 25% in humans, and is a serious public health problem in southern and eastern Asia [1,2]. It is well known that JEV infects boars and sows, which are the major amplifying hosts of JEV in nature. The treatment of JEV infection in pigs is important for controlling the prevalence of JEV in humans and economic losses in pig production. Even though two kinds of vaccines—the attenuated vaccine (SA14-14-2) and the inactivated vaccines (mouse brain-derived and Vero cell culture-derived)—are widely used to vaccinate human and pigs, JE is widespread in the south, southeast, and the east regions of Asia, with epidemics breaking out every few years [3,4]. Therefore, it is necessary to develop new strategies against JEV.

Type I interferons (IFNs, including IFN-α) mediate a wide range of biological activities, including antiviral activity, cell growth, differentiation, apoptosis, and immune response [5]. Type I IFNs bind a heterodimeric transmembrane receptor termed the IFN-α receptor to activate interferon-stimulated gene factor 3 (ISGF3) via the JAK-STAT signaling pathway and induce the coordinated upregulation of hundreds of interferon-stimulated genes (ISGs) that orchestrate an antiviral state in the cells [6]. Of these ISGs, Mx (myxovirus-resistant), PKR (Double-stranded RNA-dependent protein kinase), and OAS (2′,5′-oligoadenylate synthetases) are the three major mediators of innate antiviral mechanism induced in the host cells, and have been studied extensively. Recently, it has been shown that porcine IFN-α inhibits JEV replication [7]. Furthermore, transient overexpression of OAS isoforms inhibits JEV replication [8]. However, whether the inhibitory activity of type I IFNs on JEV is mediated by Mx proteins is largely unknown.

Mx proteins are interferon-induced dynamin-like GTPases that are present in all vertebrates [9,10,11]. These proteins have a broad range of antiviral activities against various viruses [12], such as vesicular stomatitis virus (VSV) [13,14], influenza virus [15,16], classic swine fever virus (CSFV) [17], foot mouth disease virus (FMDV) [18], and bovine viral diarrhea virus (BVDV) [19]. Mx proteins consist of an N-terminal globular GTPase domain, a connecting bundle signaling element, and the C-terminal stalk that mediates oligomerization and antiviral specificity [20]. It is well known that the dynamin-like GTPase activity—including GTP binding and GTP hydrolysis—is required for Mx to function [5,10,21]. Human MxB—which previously had not been ascribed an antiviral function—was recently found to be a suppressor of human immunodeficiency virus type 1 (HIV-1) [22,23]. Based on the nucleotide and amino acid sequences, porcine Mx1 (poMx1) has 78% homology with human MxA (huMxA) and is located in the cytoplasm of target cells, suggesting that they share similar antiviral activities against some RNA viruses. Our previous study showed that a commercial recombinant human interferon-α (huIFNα) was used to characterize the antiviral effect on JEV replication in BHK-21 cells. In this study, we sought to investigate the roles of Mx1 and Mx2 during the inhibition of JEV infection, overexpression, and knockdown of Mx1 and Mx2 were performed to determine the antiviral activities of Mx. Our findings indicate that Mx protein does not contribute to the antiviral effect of IFNα against *Flavivirus*.

## 2. Materials and Methods

### 2.1. Cells, Virus, and Interferon

The baby hamster kidney (BHK-21) cells were maintained in Dulbecco’s modified essential medium (DMEM, GIBCO, Invitrogen, Carlsbad, CA, USA) supplemented with 10% fetal bovine serum (FBS) (GIBCO, Invitrogen), 0.2% NaHCO_3_, 100 μg/mL streptomycin, and 100 IU/mL penicillin (GIBCO, Invitrogen) at 37 °C with 5% CO_2_. Porcine kidney (PK-15) cells were grown in RPMI 1640 (GIBCO, Invitrogen) supplemented with 10% FBS, 100 μg/mL streptomycin, and 100 U/mL penicillin. JEV virulent strain NJ2008 (GenBank: GQ918133) used in this study was described previously [24]. JEV attenuated vaccine strain (SA14-14-2) was purchased from Wuhan Keqian Biology Co., Ltd. (Wuhan, China). CSFV virulent strain Shimen (GenBank: AF092448) was obtained from the National Institute of Veterinary Drug Control (Beijing, China). The commercial human interferon α-1b (huIFNα) was purchased from Shenzhen Kexing Biotech Co., Ltd. (Shenzhen, China).

### 2.2. Virus Infection and Titration

Viral infection and titration were performed as previously described [25]. Briefly, cells were adsorbed with virus at the indicated multiplicity of infection (MOI) for 1 h at 37 °C, washed to remove nonadherent virus, then incubated at 37 °C. The culture supernatants were collected, and virus titers were determined by plaque-forming assay in BHK-21 cells.

### 2.3. Antibodies

Mouse anti-JEV NS1 (2B8), NS5 (1G6), and E (2A5) mAbs were kindly provided by Professor Shengbo Cao (Huazhong Agricultural University, Wuhan, China). Mouse anti-Mx1 mAb (ab79609), rabbit anti-Mx2 antibody (ab196833) and rabbit anti-Viperin antibody (ab121042) were purchased from Abcam (Cambridge, UK). Rabbit anti-green fluorescent protein (GFP) antibody was purchased from Sigma-Aldrich (St. Louis, MO, USA). Goat anti-mouse immunoglobulin G (IgG) (Alexa Fluor-568) was purchased from Thermo Fisher (Cambridge, MA, USA). Rabbit anti-β-actin mAb (13E5), goat anti-rabbit IgG-HRP (sc-2004) and goat anti-mouse IgG-HRP (sc-2005) were purchased from Santa Cruz Biotechnology (Santa Cruz, CA, USA). Stat-1 α/β rabbit polyclonal antibody was purchased from Beyotime Biotech Co., Ltd. (Nanjing, China).

### 2.4. Plasmids

pEGFP-poMx1 expressing wild-type poMx1 fused to green fluorescent protein was reported previously [17]. pcDNA3.0-poMx1 expressing wild-type poMx1 fused to HA tag was constructed from pEGFP-poMx1 using a pair of primers (pcDNA3.0-poMx1-F/R). pcDNA3.0-poTMx1 expressing a nuclear form of wild-type poMx1 [26] fused to HA tag was constructed from pEGFP-poMx1 using a pair of primers (pcDNA3.0-poTMx1-F/R). pcDNA3.0-poMx1(ΔL4) expressing a poMx1 variant (deletion of residues 534 to 573) [27] fused to HA tag was constructed by directed mutagenesis (Vazyme Biotech Co., Ltd., Nanjing, China) based on pEGFP-poMx1. Porcine Mx2 cDNA (GenBank: AB258432) was commercially synthesized by Nanjing Genscript Corporation (Nanjing, China) and cloned in-frame with EGFP into the vector pEGFP-C1 at Hind III and Xho I sites using a pair of primers (pEGFP-poMx2-F/R). pcDNA3.0-poMx2 was constructed from pEGFP-poMx2 using a pair of primers (pcDNA3.0-poMx2-F/R). Human MxA (GenBank: P20591) and mouse Mx1 (GenBank: P09922) cDNA were kindly provided by Dr. Song Gao (Sun Yat-sen University Cancer Center, Guangzhou, China) and cloned in-frame with EGFP into the vector pEGFP-C1 at Xho I and BamH I sites using two pairs of primers (pEGFP-huMxA-F/R, pEGFP-mmMx1-F/R). All of the primer pairs are listed in Table 1. The corresponding genes were verified by sequencing.

### 2.5. Immunofluorescence Assay

Cells grown on glass coverslips were infected with JEV at an MOI of 0.05. At 24 hpi, cells were washed with PBS, and fixed with 4% paraformaldehyde in PBS. Cells were then permeabilized with 0.2% Triton X-100, washed again, then reacted with either anti-JEV E, NS1, or NS5 mAbs diluted 1:500. After washing, the coverslips were reacted with goat anti-mouse IgG (Alexa Fluor-568). After washing, cells were visualized by confocal microscopy (Leica Sp5 AOBS confocal system) with a 63 _HCX PL Apo 1.4 oil immersion objective.

### 2.6. Western Blot Analysis

Cells were washed three times with PBS and lysed in cold lysis buffer (1% Triton X-100, 1 mM PMSF in PBS) for 10 min. The lysates were clarified by centrifugation at 12,000× *g* for 10 min. Total cell extracts were separated by SDS-PAGE, transferred to nitrocellulose membranes, and then probed with the indicated antibodies (anti-JEV E, NS1, or NS5 mAbs), followed by goat anti-mouse IgG-HRP conjugate antibody or goat anti-rabbit IgG-HRP conjugate antibody. β-actin was used as a loading control.

### 2.7. Brefeldin A (BFA) Treatment

BHK-21 cells (1.2 × 10^6^) were transfected with 2 μg pEGFP-poMx1 or pEGFP-poMx2. Twenty-four hours post transfection, cells were infected with JEV at an MOI of 0.05. At 12 hpi, BFA (5 μg/mL) was added to the culture medium, and incubation continued for an additional 12 h as described previously [28]. The effect of poMx1 or poMx2 in these cells was analyzed by Western blot analysis and plaque assay. In order to establish the parameters for BFA treatment, the following experiments were conducted. (i) the cytotoxic effect of BFA on BHK-21 cells was established by viability assay, as described previously [29]. Briefly, sub-confluent cell cultures grown in 96-well plates were incubated with various concentrations (0–8, 10, and 15 μg/mL) of BFA for 24 h. An MTS-based viability assay (CellTiter 96 aqueous nonradioactive cell proliferation assay from Promega (Madison, WI, USA) was conducted as recommended by the manufacturer; (ii) the dose-dependent activity of BFA was characterized, 1.2 × 10^6^ BHK-21 cells were seeded into six-well plates and infected with JEV NJ2008 at an MOI of 0.05. After virus adsorption and washing, cells were maintained in medium containing BFA at various concentrations (2.5, 5, 7.5, and 10 μg/mL) or an equivalent volume of the DMSO carrier. At 24 hpi, cell supernatants were used to determine the levels of infectious virus by plaque assay. Whole cell-culture lysates were used to determine the viral protein levels by Western blot analysis; (iii) to assess the antiviral activity of BFA over time, 1.2 × 10^6^ BHK-21 cells were seeded into six-well plates and infected with JEV strain NJ2008 at an MOI of 0.05. After virus adsorption and washing, cells were maintained in medium containing 5 μg/mL BFA or an equivalent volume of the DMSO carrier. At 13, 15, 18, and 24 hpi, cell supernatants were used to determine the levels of infectious virus by plaque assay, and whole cell-culture lysates were used to determine the viral protein levels by Western blot analysis; (iv) the antiviral activity of BFA was assessed by immunofluorescence. Briefly, the JEV-infected cells were maintained in medium containing 5 μg/mL BFA or an equivalent volume of the DMSO carrier. At 24 hpi, cells were fixed and reacted with anti-JEV E or NS5 mAbs. After washing, cells were visualized by confocal microscopy (Leica Sp5 AOBS confocal system) with a 63_HCX PL Apo 1.4 oil immersion objective.

### 2.8. Knockdown Experiments

siRNA experiments were carried out in six-well plates containing BHK-21 cells or PK-15 cells starting at 2.5 × 10^5^ cells/well. siRNAs for siMx1 (sc-45260), siMx2 (sc-45261), and the negative-control siRNA (sc-37007) (Santa Cruz Biotechnology) were transfected at a concentration of 100 nM into cells using Lipofectamine 3000 (Invitrogen) according to the manufacturer’s instructions. Six hours after transfection, the medium was aspirated, fresh complete medium containing 200 ng/mL huIFNα was added, and incubation continued for an additional 12 h [30]. Cells were washed with PBS, and infected with JEV at an MOI of 0.05. Cell supernatants were used to determine the levels of infectious virus by plaque assay. Whole cell-culture lysates were used to determine viral protein levels by Western blot analysis.

### 2.9. RT-qPCR

Viral RNA was extracted from each sample using TRIzol reagent (Invitrogen). JEV and CSFV RNA loads were measured using RT-qPCR assay, as described previously [7,31].

### 2.10. Statistical Analysis

All data were presented as means ± standard deviation (S.D.) as indicated. Student’s *t*-test was used to compare the data from pairs of treated or untreated groups. Statistical significance was indicated as ns (*p* > 0.05), * (*p* < 0.05), and ** (*p* < 0.01). All statistical analyses and calculations were performed using GraphPad Prism 5 (GraphPad Software Inc., La Jolla, CA, USA).

## 3. Results

### 3.1. Mx Proteins Were Not Detectable in JEV-Infected Cells

**Figure 1:**
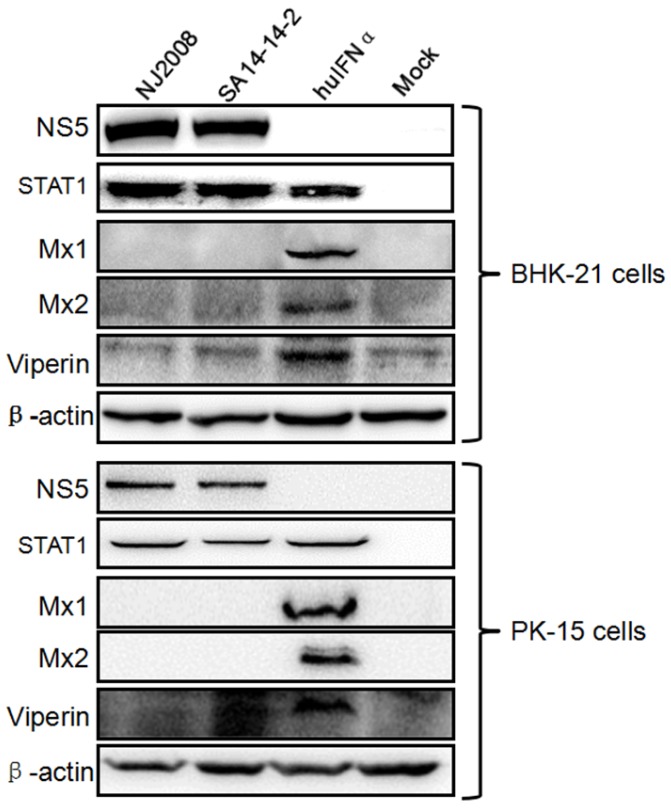
Japanese encephalitis virus (JEV) inhibits the production of interferon-stimulated gene (ISG) proteins. Cells were infected with JEV NJ2008 or SA14-14-2 strain at a multiplicity of infection (MOI) of 0.05. Alternatively, cells were treated with 200 ng/mL human interferon-α (huIFNα) for 12 h at 37 °C. After these treatments, ISG proteins in cell lysates were probed with the indicated antibodies using Western blot analysis.

Flaviviruses have evolved specific strategies to avoid and/or attenuate induction of IFN and its effector responses. In JEV, the N-terminal 83 residues of NS5 inhibit JAK-STAT signaling through a protein-tyrosine phosphatase-dependent mechanism, resulting in suppressed expression of a wide variety of interferon-stimulated genes [32]. Here, the innate immune response of the host cells against JEV infection was determined by Western blot analysis using the antibodies against ISG proteins Mx1, Mx2, and Viperin. As shown in Figure 1, endogenous Mx1, Mx2, and Viperin proteins were produced in huIFNα-treated BHK-21 and PK-15 cells (lane 3); in contrast, no endogenous Mx1, Mx2, or Viperin proteins were detectable in cells infected with virulent (NJ2008) or attenuated JEV (SA14-14-2). STAT1 expression was observed after viral infection, suggesting that the IFN-induced JAK-STAT signaling was not blocked. These data are consistent with previous studies [32] demonstrating that JEV NS5 expression hijacks STAT1 protein and blocks its nuclear translocation, causing loss of endogenous interferon-induced proteins.

### 3.2. Exogenous Mx Proteins Have No Anti-JEV Activity in Infected Cells

**Figure 2:**
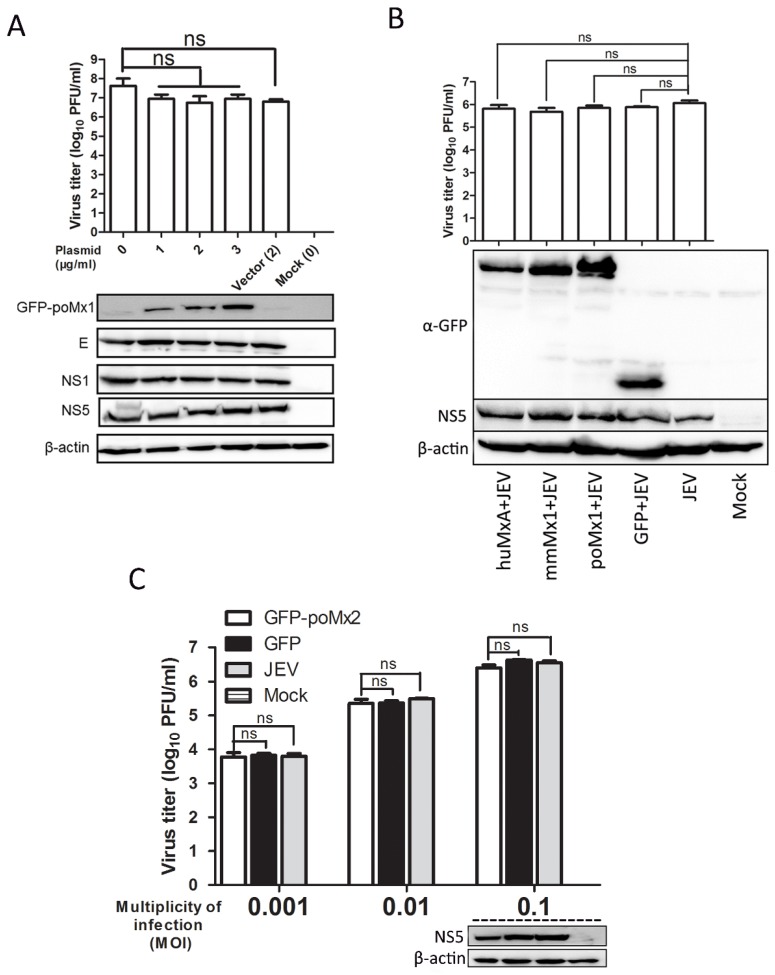
Exogenous Mx proteins fused to green fluorescent protein (GFP) have no anti-JEV activity. Three separate experiments were performed to assess the anti-JEV activity of the fused Mx1 and Mx2 protein. Cells were transfected with the constructs and then infected with JEV. At 24 hpi, lysates of the harvested cell culture were used to determine viral protein levels by Western blot analysis, the cell supernatants were used to determine the levels of infectious virus by plaque assay. (**A**) Cells transfected with the various concentrations of pEGFP-poMx1 and then infected with JEV at an MOI of 0.05; (**B**) Cells transfected with pEGFP-huMxA (human MxA), pEGFP-mmMx1, pEGFP-poMx1 (porcine Mx1), or pEGFP-C1 then infected with JEV at an MOI of 0.05; (**C**) Cells transfected with pEGFP-poMx2 or pEGFP-C1 and then infected with JEV at an MOI of 0.001, 0.01, and 0.1. All data are presented as means ± standard deviation (S.D.) as indicated. Statistical significance is indicated as ns (*p* > 0.05).

**Figure 3:**
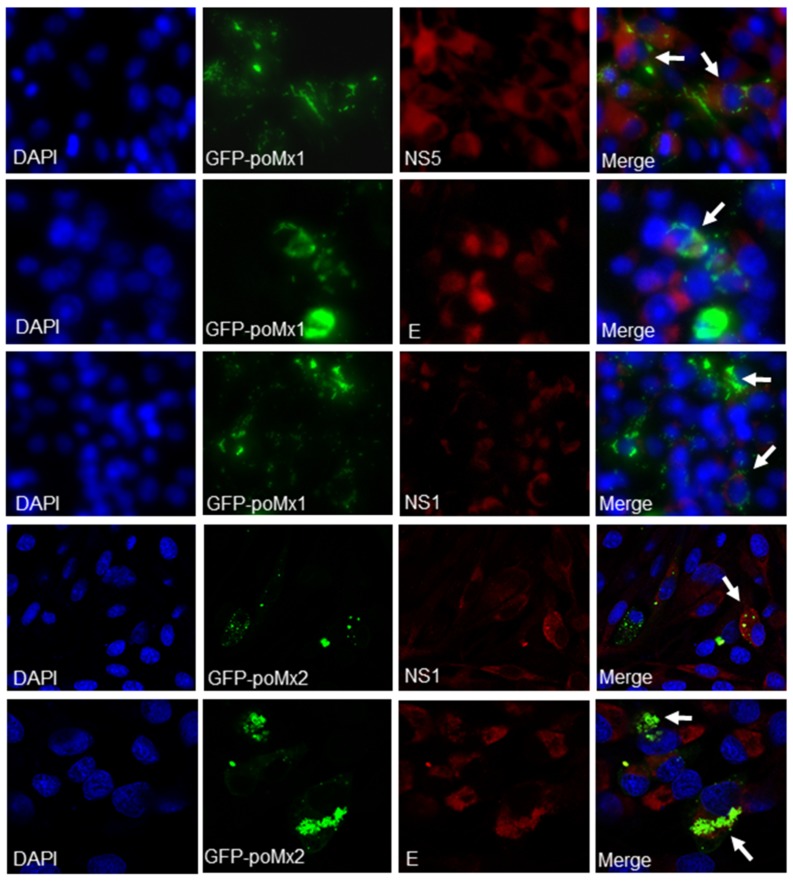
JEV replication in pEGFP-poMx1 or pEGFP-poMx2-transfected BHK-21 cells. Transfected cells grown on glass coverslips were infected with JEV at an MOI of 0.05. At 24 hpi, cells were washed with PBS and subjected to immunofluorescence assay. The cell nucleus was counterstained with DAPI (blue). White arrows indicate cells with high GFP-poMx1 or GFP-poMx2 expression levels along with high JEV viral proteins (NS1, NS5, or E).

Previous reports have shown that exogenous Mx proteins inhibit the replication of a wide range of viruses [12]. In this study, poMx1 and poMx2 were over-expressed in BHK-21 or PK-15 cells, and JEV replication was assessed. First, BHK-21 cells were transfected with various concentrations of pEGFP-poMx1 and infected with JEV at an MOI of 0.05. At 24 hpi, the effect of GFP-poMx1 on JEV replication was analyzed by Western blot analysis and plaque assay. Although the expression level of the GFP-poMx1 fusion protein increased with increasing construct concentration, the level of viral proteins E, NS1, and NS5 were comparable in all transfected cells, and roughly equal amounts of progeny virus were produced (Figure 2A). The Mx protein family is highly conserved, and similar results were obtained with JEV-infected cells over-expressing pEGFP-huMxA or pEGFP-mmMx1. As shown in Figure 2B by plaque assay and Western blot analysis, viral titers and NS5 protein expression were roughly equal for each construct-transfected sample. These data demonstrate that JEV replication is not inhibited by over-expression of GFP-poMx1, huMxA, or mmMx1. Similar experiments were performed to evaluate Mx2 antiviral activity. BHK-21 cells were transfected with the pEGFP-poMx2 construct and infected with JEV at MOI 0.001, 0.01, and 0.1. As shown in Figure 2C, JEV replication in pEGFP-poMx2-transfected cells was the same as that in pEGFP-C1-transfected cells, suggesting that GFP-poMx2 has no direct anti-JEV activity. Immunofluorescence assays were performed to assess JEV replication in pEGFP-poMx1- or pEGFP-poMx2-transfected cells. As shown in Figure 3, red fluorescence—indicating viral proteins—was observed in GFP-poMx1 and GFP-poMx2-positive cells (indicted by green fluorescence). These data demonstrate that exogenous porcine Mx1 and Mx2, human MxA, and mouse Mx1 proteins fused to GFP had no obvious anti-JEV activity.

**Figure 4:**
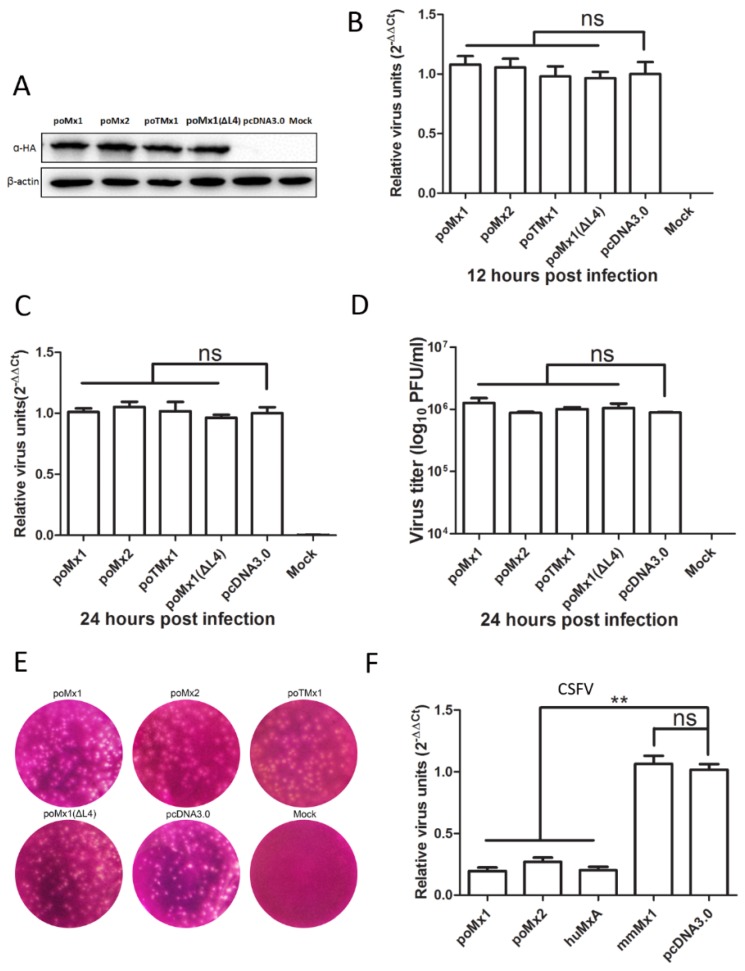
The different isoforms of porcine Mx proteins have no anti-JEV activity. (**A**) Western blot analysis of the lysates of PK-15 cells transfected with the indicated constructs. Overexpression of the different porcine Mx proteins was determined using anti-HA Flag mouse monoclonal antibody; (**B**,**C**) PK-15 cells were transfected with these constructs and infected with JEV at an MOI of 0.05. At 12 and 24 hpi, JEV replication was analyzed by RT-qPCR; (**D**,**E**) At 24 hpi, JEV replication was analyzed by plaque assay; (**F**) Classic swine fever virus (CSFV) replication (positive control) was inhibited by some exogenous Mx proteins. All data are presented as means ± standard deviation (S.D.) as indicated. Statistical significance is indicated as ns (*p* > 0.05) and ** (*p* < 0.01).

To address the possible influence of GFP on the function of Mx proteins, we constructed another set of plasmids as follows: pcDNA3.0-poMx1, pcDNA3.0-poMx2, pcDNA3.0-poTMx1 and pcDNA3.0-poMx1(ΔL4). PK-15 cells were transfected with these constructs and infected with JEV at an MOI of 0.05. At 12 and 24 hpi, JEV replication was analyzed by Western blot analysis, RT-qPCR, and plaque assay. As shown in Figure 4, at 12 and 24 hpi, JEV RNA levels were roughly equal among the cells overexpressing the different isoforms of porcine Mx, suggested that none of the isoforms affected JEV replication. Plaque assay data showed that viral titers in cells overexpressing Mx proteins were the same as that in the control cells. However, as a positive control, we saw that CSFV replication was inhibited in cells overexpressing poMx1, poMx2, and huMxA, but not mmMx1. This is consistent with previous studies [17,33]. Overall, exogenous poMx1 or poMx2 had no demonstrable anti-JEV activity.

### 3.3. Mx1 or Mx2 Depletion Did Not Affect the Antiviral Activity of IFN

**Figure 5:**
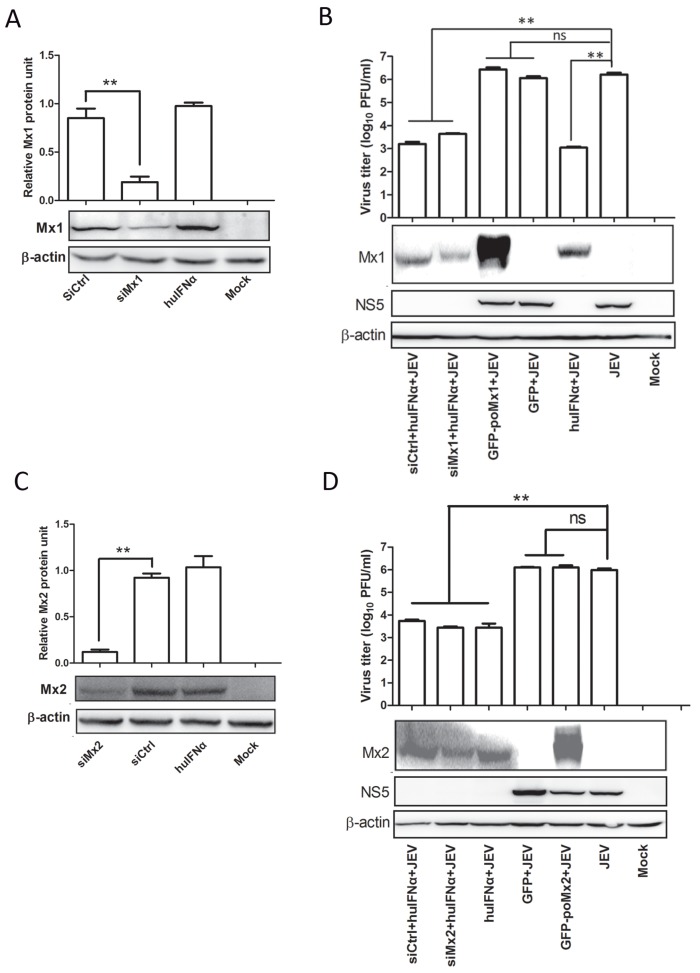
IFNα inhibits JEV replication in Mx-knockdown BHK-21 cells. Cells were transfected with siRNA targeting Mx1, Mx2, and a siRNA control (siCtrl). Six hours after transfection cells were treated with huIFNα for 12 h, then infected with JEV at an MOI of 0.05. At 24 hpi, cell supernatants were used to determine the levels of infectious virus by plaque assay, and the cell culture lysates were used to determine the viral protein levels by Western blot analysis. Percent knockdown of (**A**) Mx1 and (**C**) Mx2. JEV replication determined by plaque assay and Western blot analysis in (**B**) siMx1- or (**D**) siMx2-transfected and control cells. Quantification of the blotted proteins was performed using Image J software. All data are presented as means ± standard deviation (S.D.) as indicated. Statistical significance is indicated as ns (*p* > 0.05) and ** (*p* < 0.01).

To determine whether Mx expression is necessary to inhibit JEV replication, endogenous Mx1 or Mx2 was knocked down in interferon-treated BHK-21 cells and PK-15 cells prior to virus infection. Cells were transfected for 6 h with the commercial Mx1, Mx2 siRNA, or negative control siRNA (siCtrl), then treated with 200 ng/mL of huIFNα for 12 h. Mx1 expression was reduced by 66% in cells transfected with Mx1 siRNA compared to the negative control (Figure 5A). Subsequently, these siRNA-transfected-huIFN-treated cells were infected with JEV at an MOI of 0.05, and viral protein expression levels and virus titers were assessed. NS5 levels were roughly equally suppressed in siCtrl- and siMx1-transfected cells and non-transfected control cells, and significantly suppressed compared to untreated controls. Likewise, virus titers in these samples were roughly equal and significantly reduced compared to untreated controls (Figure 5B). These data demonstrate that knockdown of endogenous Mx1 does not impair the antiviral ability of IFN. Mx2 expression was reduced by 79% in cells transfected with Mx2 siRNA compared to the negative control (Figure 5C), and results similar to those described above were observed in huIFNα-treated cells with Mx2 knockdown. NS5 levels were suppressed in siCtrl- and siMx2-transfected cells similar to that in huIFNα-treated cells transfected with siCtrl and siMx1, and significantly suppressed compared to untreated controls. Virus titers in these samples were roughly equal and significantly reduced compared to untreated controls (Figure 5D). These data demonstrate that knockdown of endogenous Mx2 does not impair the antiviral ability of IFN.

**Figure 6:**
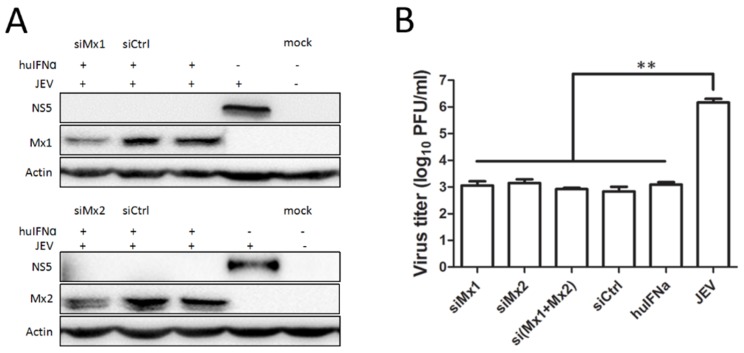
IFNα inhibits JEV replication in Mx-knockdown PK-15 cells. Mx1 and Mx2 knockdown in PK-15 cells was performed as described above. At 24 hpi, lysates of cell culture were used to determine the viral protein levels by Western blot analysis (**A**), and cell supernatants were used to determine the levels of infectious virus by plaque assay (**B**). All data are presented as means ± standard deviation (S.D.) as indicated. Statistical significance is indicated as ** (*p* < 0.01).

Similar siRNA experiments were performed in PK-15 cells. After the successful knockdown of endogenous porcine Mx1 or Mx2 as described above, the levels of NS5 (Figure 6A) and virus titers (Figure 6B) were significantly reduced in huIFNα-treated cells compared to the controls, which suggests that IFN can effectively inhibit JEV replication by an Mx-independent pathway.

### 3.4. Mx Does Not Inhibit JEV Replication in Cells Treated with BFA

**Figure 7:**
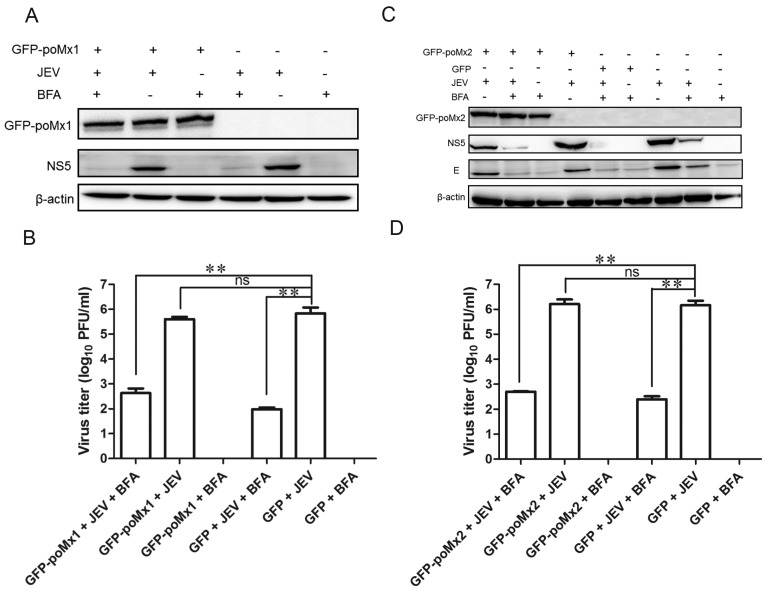
Mx1 or Mx2 overexpression has no impact on JEV replication in cells treated with Brefeldin A (BFA). GFP-poMx1 (or poMx2)-overexpressing BHK-21 cells and control cells were infected with JEV at an MOI of 0.05. At 12 hpi, BFA (5 μg/mL) was added to the culture medium for an additional 12 h. The lysates of cell culture were used to determine the viral protein levels by Western blot analysis, and cell supernatants were used to determine the levels of infectious virus by plaque assay. Western blot analysis (**A**) and virus titer (**B**) of infectious virus of GFP-poMx1-overexpressing infected cells treated with or without BFA. Alternatively, Western blot analysis (**C**) and virus titer (**D**) of infectious virus of the GFP-poMx2-overexpressing infected cells treated with or without BFA. All data are presented as means ± standard deviation (S.D.) as indicated. Statistical significance is indicated as ns (*p* > 0.05) and ** (*p* < 0.01).

BFA is a Golgi apparatus-disrupting agent which prevents the development of virus-induced membranes when added before the end of the latent period of *Flavivirus* infection [34]. A previous report showed that West Nile Virus (WNV) replication was significantly reduced in BFA-treated Vero cells overexpressing huMxA, compared to BFA-treated Vero cells, suggesting that WNV-induced membranes may provide partial protection against huMxA [28]. Here, we performed a series of experiments to examine whether a similar mechanism exists in JEV infection. Initially, we confirmed that BFA treatment disrupted the Golgi apparatus by visualizing giantin (Golgi marker) in BFA-treated and untreated cells using the anti-giantin antibody. Subsequently, GFP-poMx1 (or poMx2)-overexpressing BHK-21 cells and mock-transfected cells were infected with JEV at an MOI of 0.05. At 12 hpi, BFA (5 μg/mL) was added to the culture medium for an additional 12 h, as described previously. As shown in Figure 7A,C, in the absence of BFA, GFP-poMx1- (or poMx2)-overexpressing cells, and mock-transfected cells showed comparable levels of NS5 expression, as expected. However, in the presence of BFA, NS5 and E expression was reduced significantly, with no statistical difference between GFP-poMx1- (or poMx2) transfected cells and mock-transfected cells. Virus titers were also decreased significantly (Figure 7C,D). These data indicated that BFA—not Mx—inhibits JFV replication. Collectively, these results demonstrated that poMx1 or poMx2 inhibit JEV replication in the presence of BFA, suggesting that JEV is resistant to IFN-induced Mx protein via an unknown pathway.

### 3.5. BFA Effectively Inhibits JEV Replication

**Figure 8:**
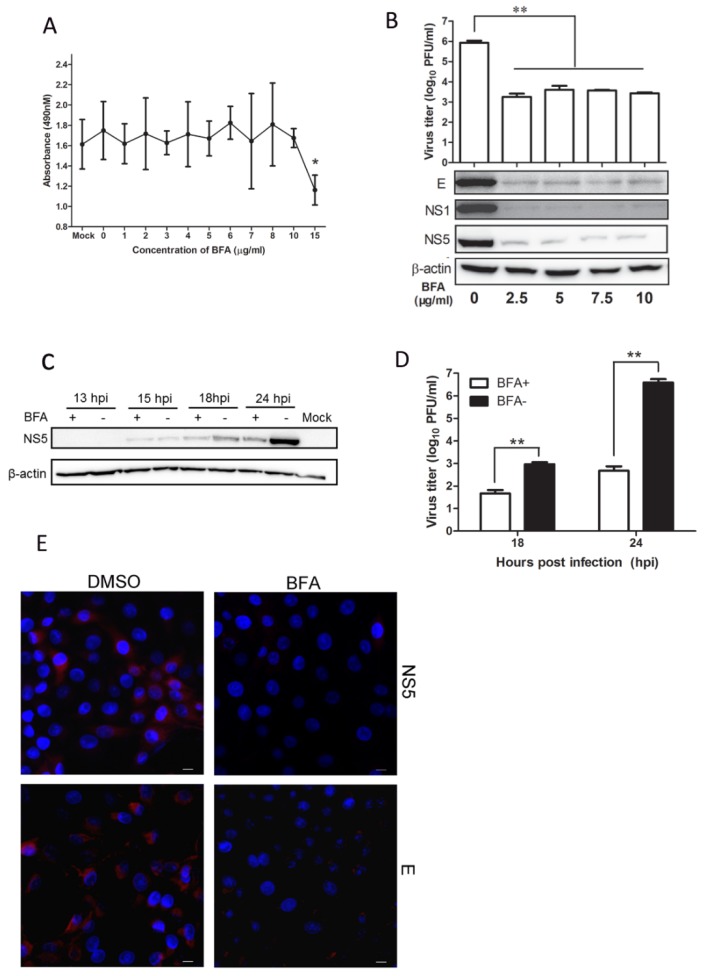
Antiviral activity of BFA. (**A**) Cytotoxic effect of BFA. BHK-21 cells at 80% confluence in 24-well plates were treated with various concentrations of BFA for 24 h. After treatment, a cell proliferation reagent was added to each well, and 2 h later, the absorbance at 490 nm was recorded. (**B**–**E**) BHK-21 cells were seeded into six-well plates and infected with JEV at an MOI of 0.05. After virus adsorption and washing, cells were maintained in medium containing BFA at various concentrations or an equivalent volume of DMSO. At 24 hpi, cell culture lysates were used to determine viral protein levels by Western blot analysis, cell supernatants were used to determine levels of infectious virus by plaque assay (**B**); Infected cells were maintained in medium containing BFA at 5 μg/mL or an equivalent volume of DMSO. At 13, 15, 18, and 24 hpi, cell culture lysates were used to determine viral protein levels by Western blot analysis (**C**); Cell supernatants were used to determine levels of infectious virus by plaque assay (**D**); The inhibitory effect of BFA was detected using anti-JEV NS5 or E mAbs by confocal microscopy (**E**). JEV was strained with red fluorescence, and nucleus was strained with DAPI. All data are presented as means ± standard deviation (S.D.) as indicated. Statistical significance is indicated as ** (*p* < 0.01).

To further explore the antiviral activity of BFA, we performed a series of experiments as follows. Cells were treated with 0 to 15 μg/mL BFA for 24 h, and the cytotoxic effect was evaluated to ensure the sub-toxic doses of BFA. As shown in Figure 8A, cells tolerated up to 10 μg/mL BFA. The cell viability was reduced only slightly in the presence of 15 μg/mL BFA. To test the effects of BFA on JEV production, cells were infected with JEV and then treated with various concentrations of BFA. At 24 h post treatment, the viral protein levels of E, NS1, and NS5 in BFA-treated cells were significantly reduced compared to that in DMSO-treated cells, which is consistent with the decreasing of virus titer. The results showed that virus titer was reduced by about 209-fold, suggesting that BFA up to 2.5 μg/mL inhibits JEV replication in a dose-independent manner (Figure 8B). To test whether BFA inhibits JEV replication in a time-dependent manner, cells were infected with JEV at an MOI of 0.05 and then treated with 5 μg/mL BFA. At 13, 15, 18, and 24 hpi, the viral protein levels in lysed cells were determined by Western blot analysis, and the amount of infectious virus in cell supernatants was determined by plaque assay. The results showed that NS5 protein level in the BFA-treated cells reduced significantly by 18 hpi when compared to untreated cells (Figure 8C). Plaque numbers were in accord with the results above, and virus titers at 18 and 24 hpi were reduced by 20-fold and 8175-fold, respectively (Figure 8D), suggesting that BFA strongly inhibits JEV replication in a time-dependent manner. The inhibitory effect of BFA was analyzed by immunofluorescence assay. The results showed that viral protein levels were significantly decreased at 24 hpi (as indicated by red fluorescence) compared to untreated cells (Figure 8E), suggesting that JEV replication was strongly inhibited by BFA.

## 4. Discussion

Type I interferon (IFN) is abundantly produced in virus-infected cells soon after infection, as well as a myriad additional virus-initiated modulatory effects, including induction of cellular inhibitors or repressors of transcription, and activation of IFN-I stimulated genes (ISG) and proteins, all in order to antagonize an antiviral host response [35]. Thus, IFNs have been used as antiviral agents in the treatment of several pathogens, including Flaviviridae [36]. Previous reports have shown that IFNα has activity against JEV in PK-15 cells [7,8] and BHK-21 cells [37]. In addition, IFNα and IFNƔ can both efficiently prevent WNV infection, though IFNα demonstrated the greater antiviral efficacy [38]. Furthermore, the ISGs induced by interferons inhibit WNV and DENV replication at different stages through different mechanisms [39,40,41]. Although type I IFNs—IFN-α/β—are important innate immune regulators for resisting viral infections, it has been demonstrated that flaviviruses produce effective immune modulatory proteins and utilize multiple immune evasion mechanisms that limit host immune responses and advance viral replication. Previous reports have shown that JEV and DEV NS5 protein is an IFN antagonist and that it may play a role in blocking IFN-stimulated JAK-STAT signaling via activation of PTPs during JEV infection, resulting in suppression of the expression of a wide variety of ISGs which can establish antiviral, anti-proliferative, and/or immune-regulatory states in host cells [32,42]. In this study, BHK-21 cells and PK-15 cells were infected with JEV virulent strain NJ2008 or attenuated vaccine strain SA14-14-2. Figure 1 showed that JEV-infected cells did not produce endogenous Mx1, Mx2, or Viperin above background levels, suggesting that the production of IFN-induced ISGs were suppressed by JEV. Our findings are consistent with the recent report that a low-level induction of IFNα mRNA expression was observed after JEV infection [8].

ISGs are the key antiviral factors in the tug-of-war between interferons and JEV, and the different ISGs have been demonstrated to be induced by different stimuli [43]. To date, previous reports have shown that ISG15, Viperin, and OAS have anti-JEV activities in the respective manners. Overexpression of ISG15 significantly reduced the JEV-induced cytopathic effect and inhibited JEV replication by activating the expression of STAT1-dependent genes including IRF-3, IFN-β, IL-8, PKR, and OAS before and post-JEV infection [44]. In addition, although the antiviral activities of porcine OAS1, OAS2, and OSAL against JEV were demonstrated in PK-15 cells, their antiviral mechanisms need further investigation [8]. Overexpression of Viperin significantly decreased the production of JEV in the presence of the proteasome inhibitor MG132 that sustained Viperin levels [45]. Mx proteins—the IFN-induced GTPase—are key components of the antiviral state induced by interferons in many species. Our previous work showed that overexpression of porcine Mx1 could inhibit CSFV replication in vitro and in vivo [13,17], as well as VSV replication [31]. Here, we explore the role of Mx proteins during IFNα-inhibited JEV infection. Unexpectedly, even though IFNα effectively blocks JEV infection, Mx proteins play no apparent role inhibiting JEV replication. Overexpression of Mx isoforms including porcine Mx1 and Mx2, human Mx1, and mouse Mx1 did not inhibit JEV replication as determined by Western blot analysis, plaque assay, and immunofluorescence assay. Furthermore, we found that huIFNα still inhibited JEV replication where Mx1 or Mx2 was knocked down using RNA interference, suggesting that Mx is not a critical factor in the pathway whereby IFN inhibits JEV infection. Previous studies of the overexpression of MxA in Vero cells showed WNV_KUN_ replication, maturation, and secretion was uninhibited [28]. However, retargeting MxA expression from cytoplasmic inclusions to the endoplasmic reticulum during WNV_KUN_ replication did significantly hamper the formation and spread of infectious WNV_KUN_ virions [46]. We speculated that JEV may be resistant to Mx protein through a similar mechanism.

BFA has been widely used to study membrane trafficking and protein processing in eukaryotic cells [47,48]. After the treatment of mammalian cells with BFA, ER to Golgi transport is rapidly inhibited. Previous reports have shown that BFA acts in a variety of ways as an antiviral, such as arresting the maturation and egress of herpes simplex virus particles during infection [49] and inhibiting *Pestivirus* release from infected cells without affecting its assembly and infectivity [50]. In addition, short-term (1 h) BFA treatment inhibits VSV gene expression, while long-term (12 h) treatment blocks VSV entry [51]. BFA completely inhibits poliovirus RNA synthesis by preventing the formation of secretory vesicles [52,53]. This is the first report that BFA harbors anti-JEV activity. We found that BFA treatment at low concentration effectively inhibits JEV proliferation in a dose-independent and time-dependent manner. Because overexpression of human MxA inhibits WNV replication in the presence of BFA [28], we hypothesized that BFA targets a similar mechanism in JEV-infected cells. Unexpectedly, we found overexpression of porcine Mx1 or Mx2 did not inhibit JEV replication in the presence of BFA, clearly, understanding its antiviral mechanisms against JEV at the cellular level needs further study. Our data suggests that it would be feasible to develop BFA as a potential reagent against JEV infection.

Taken together, the data from overexpression and knockdown of Mx proteins have indicated that IFNα inhibits JEV replication by Mx-independent pathway. Moreover, JEV-induced membranes did not provide any protection against Mx protein in the presence of BFA. That BFA can inhibit JEV replication suggests that BFA could be developed into an antiviral reagent.

## Figures and Tables

**Table 1:** Primers used in this study.

Primer	Sequence (5′ → 3′)
pEGFP-poMx2-F	TGACAAGCTTACCATGCCTAAACCCCGCATGTCG
pEGFP-poMx2-R	TGACCTCGAGTTACCCCTGTAATGACTGAGC
pcDNA3.0-poMx2-F	TGACAAGCTTACCATGCCTAAACCCCGCATGTCG
pcDNA3.0-poMx2-R	TGACCTCGAGTTAAGCGTAGTCTGGGACGTCGTATGGGTAC CCCTGTAATGACTGAGC
pEGFP-huMxA-F	TGACCTCGAGCTACCATGGTTGTTTCCGAAGTGGACATC
pEGFP-huMxA-R	TGACGGATCCACCGGGGAACTGGGCAAGCCGGCG
pEGFP-mmMx1-F	TGACCTCGAGCTACCATGGATTCTGTGAATAATCTGTGC
pEGFP-mmMx1-R	TGACGGATCCATCGGAGAATTTGGCAAGCTTCTG
pcDNA3.0-poMx1-F	TGACAAGCTTACCATGGTTTATTCCAGCTGTG
pcDNA3.0-poMx1-R	TGACCTCGAGTTAAGCGTAGTCTGGGACGTCGTATGGGTAGC CTGGGAACTTGGCGA
pcDNA3.0-poTMx1-F	TGACAAGCTTACCATGGACAAGGAGTTCCTGGAGGCTCCTAA GAAGAAGAGAAAGGTTGAGTTCAGAATTGTTTATTCC
	AACTGTGAAAGTAAAGAACCTGATTCAGTT
pcDNA3.0-poTMx1-R	TGACCTCGAGTTAAGCGTAGTCTGGGACGTCGTATGGGTAGCC TGGGAACTTGGCGA
